# Damage and repair: How *Poaceae* plants fix DNA damaged by UV-B radiation

**DOI:** 10.1093/plphys/kiae094

**Published:** 2024-02-20

**Authors:** Maneesh Lingwan

**Affiliations:** Plant Physiology, American Society of Plant Biologists; Donald Danforth Plant Science Center, St. Louis, MO 63132, USA

Plants rely on sunlight for the process of photosynthesis, but being sessile they are prone to detrimental UV-B (290-320 nm) radiation, which can potentially disrupt their macromolecules, including lipids, proteins, and membranes (Yadav et al. 2020). Furthermore, UV-B causes DNA damage by generating cyclobutane pyrimidine dimers (CPDs) that impede the replication and transcription processes.

DNA repair is crucial for all organisms to ensure their survival and genomic integrity. Photoreactivation is a unique and efficient means of reversing DNA damage. It is conserved in a diverse range of species, including plants, and is carried out by an enzyme called photolyase (PHR) (Harm 1976; Takahashi et al. 2011). PHR uses blue and UV-A rays (315-400 nm) to generate energy for monomerizing dimers, facilitating the restoration of damaged molecules. Studies have shown that Arabidopsis and rice (*Oryza sativa*) plants lacking PHR display a hypersensitive response to UV-B radiation (Mmbando et al. 2020). Rice CPD photolyase (OsPHR) is encoded by a single-copy gene, and the encoded protein accumulates in the nucleus, mitochondria, and chloroplasts. Prior studies have reported nuclear and mitochondrial targeting sequences are at the C terminus of the protein; however, the precise positions and characteristics of the chloroplast-targeting information in OsPHR are still unidentified (Takahashi et al. 2014).

In this issue of *Plant Physiology*, Otake et al. (2024) identified the key sequences accountable for the transport of OsPHR to chloroplasts. The sequence at the N-terminal region of OsPHR was analyzed to predict chloroplast-targeting sequences using the TargetP-2.0 (Almagro Armenteros et al. 2019). The sequence analysis suggested that there is an N-terminal signal peptide capable of translocating the protein to the endoplasmic reticulum (ER) but did not reveal an obvious chloroplast-targeting sequence. However, their functional studies indicated that the N-terminal sequence is necessary for OsPHR to move into the chloroplasts, leading them to investigate this process further.

It was previously reported that some other chloroplast-localized proteins do not possess the typical chloroplast-targeting transit peptides, but instead are translocated via the ER-Golgi system (Kleffmann et al. 2004). To investigate whether this is the case for OsPHR, Otake et al. (2024) disrupted Golgi-mediated vesicular traffic with Brefeldin A, which impeded the movement of OsPHR into chloroplasts and thus revealed that this pathway is used for OsPHR movement into chloroplasts. The N-terminal hydrophobic region of OsPHR includes several proline residues. A transient expression vector was constructed in which each proline residue in the 1 to 14 amino acid region was substituted with an alanine. The movement of OsPHR to the chloroplasts was significantly impeded after the substitution of proline residues with alanine, indicating that the translocation of OsPHR across the chloroplast membrane is facilitated by proline residues.

Previous studies indicated that in its phosphorylated form, OsPHR is not efficiently transported into the chloroplasts but rather enters the nucleus or mitochondria (Teranishi et al. 2008; Takahashi et al. 2014). The N-terminal region of OsPHR has several serine residues that are likely to be phosphorylation sites. Otake et al. (2024) substituted the seventh serine residue of OsPHR with an alanine residue to produce an unphosphorylated form and replaced it with an aspartic acid or a glutamic acid residue to produce a pseudo-phosphorylated form of OsPHR. These studies showed that translocation of OsPHR into the chloroplasts occurs when the seventh serine residue is unphosphorylated, but when it is phosphorylated, it moves into the nucleus and mitochondria (Fig. 1), consistent with previous studies.

**Figure 1.**
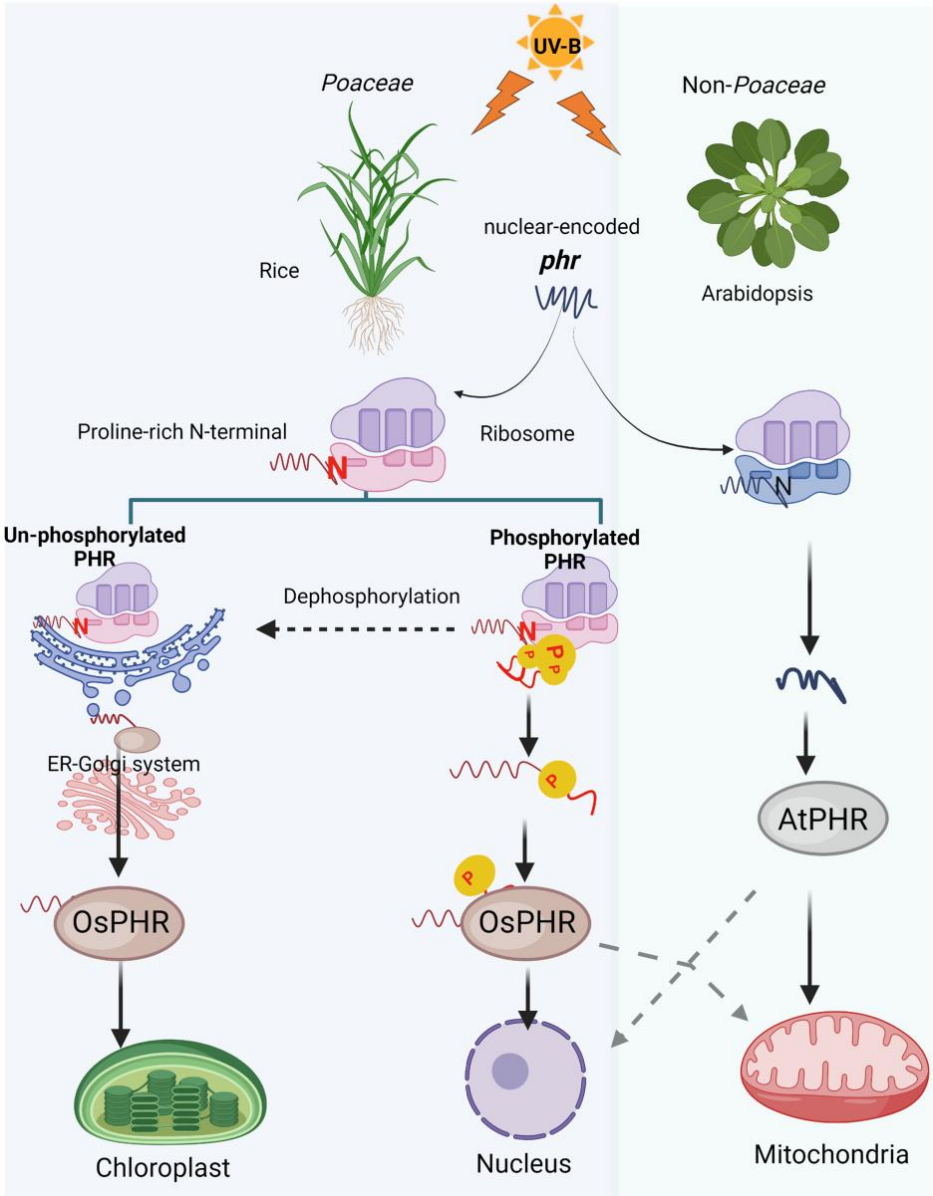
Model shows how rice PHR is transported from the ER to the Golgi apparatus and then to the chloroplasts. Nuclear-encoded PHR proteins that are transported to chloroplasts through the ER-Golgi system have a vesicular signal peptide in their N-terminal region that is essential for the movement of OsPHR to the chloroplast. PHR is not transported to the chloroplast when phosphorylated; instead, it goes to the mitochondria and the nucleus. The PHR proteins from non-*Poaceae* species, for example, AtPHR, are not transported to chloroplasts. The N-terminal sequence is marked in red and has a high proportion of proline residues. A yellow circle with the letter P denotes the phosphorylated serine. Model adapted from Otake et al. (2024) and recreated using BioRender.

Because the sequence of amino acids at position 1 to 14 of OsPHR is not present in other PHR proteins, such as those from *Arabidopsis thaliana* (AtPHR), Otake et al. (2024) analyzed the transient expression of AtPHR in rice protoplasts. The findings revealed that rice protoplasts expressing AtPHR did not show fluorescence in the chloroplasts, indicating that AtPHR does not undergo translocation or localization to chloroplasts in Arabidopsis. However, the PHR proteins of other members of the Poaceae family, such as wheat (*Triticum aestivum*) and barley (*Hordeum vulgare*), do translocate to the chloroplast. The results suggested that the ability of PHR to specifically target chloroplasts may be restricted to plants belonging to the *Poaceae* family. Nevertheless, the underlying mechanism behind these findings remains unknown.

Transgenic rice plants that do not have PHR activity in their chloroplasts show leaf browning when exposed to UV-B radiation. This suggests that in rice, PHR has to be active within chloroplasts to protect plants from UV-B radiation (Otake et al. 2024). Rice and other *Poaceae* may have developed the ability to transport PHR independently to chloroplasts to repair CPD in chloroplast DNA. These variations indicate that plants exhibit variability in their resistance and adaptation mechanisms to cope with UV-B radiation. The different mechanisms of DNA repair in chloroplasts may have more significance in *Poaceae* species cultivated under high solar radiation than in plants that thrive under low-radiation settings. Exploring the translocation mechanism of PHRs in other plant species will be a fascinating opportunity in the future to uncover novel UV-B protection and adaptation strategies.

## Dive Curated Terms

The following phenotypic, genotypic, and functional terms are of significance to the work described in this paper:

CPD Gramene: AT5G05690
CPD Araport: AT5G05690

dna Gramene: EO:0007532
dna Araport: EO:0007532
